# Case Report: Palliative radiotherapy monitoring using computed tomography and diffusion-weighted magnetic resonance imaging in a cat with ceruminous gland adenocarcinoma

**DOI:** 10.3389/fvets.2025.1682168

**Published:** 2025-10-27

**Authors:** Junyoung Kim, Soyeon Kim, Sunghwa Hong, Eunjee Kim, Junghee Yoon, Jihye Choi

**Affiliations:** ^1^College of Veterinary Medicine and Veterinary Medical Research Institute, Jeju National University, Jeju, Republic of Korea; ^2^College of Veterinary Medicine and The Research Institute for Veterinary Science, Seoul National University, Seoul, Republic of Korea

**Keywords:** ear canal tumor, feline, palliative radiation therapy, ceruminous adenocarcinoma, apparent diffusion coefficient

## Abstract

**Background:**

Ceruminous gland adenocarcinoma is the most common malignant tumor of the feline external ear canal. These tumors are often locally invasive and may extend into adjacent musculature and lymph nodes, limiting the feasibility of surgical excision. When surgery is not an option, radiation therapy can provide palliative benefit. Advanced imaging modalities, including computed tomography (CT) and magnetic resonance imaging (MRI), play a crucial role in treatment planning and therapeutic monitoring.

**Case description:**

A 14-year-old spayed female Persian cat with a confirmed diagnosis of ceruminous gland adenocarcinoma presented with a progressively enlarging mass in the left external ear canal. Due to extensive local invasion into the adjacent parotid and salivary regions, surgical resection was not feasible. The cat was treated with palliative-intent radiation therapy (36 Gy in 6 weekly fractions). Serial CT and MRI, including diffusion-weighted imaging (DWI) and apparent diffusion coefficient (ADC) mapping, were performed on days 0 (radiotherapy completion), 30, 90, and 180 to monitor treatment response. During the initial follow-up period, imaging demonstrated sustained tumor size reduction, symptomatic improvement, and minimal adverse effects. Functional MRI delineated residual lesions more clearly than conventional sequences, supporting its role in post-treatment assessment. However, on day 180, imaging revealed suspected local recurrence and regional lymph node metastases, suggesting disease progression. Despite this, the treatment successfully achieved the intended palliative goals, including temporary tumor control and clinical stabilization.

**Conclusion:**

This report described a rare case of feline ceruminous gland adenocarcinoma characterized by local invasiveness that precluded surgical resection. Palliative radiotherapy was administered, and sequential imaging using computed tomography and diffusion-weighted magnetic resonance imaging, including ADC mapping, enabled detailed monitoring of the tumor response. Although conventional imaging confirmed size reduction, DWI-MRI better delineated residual lesions, suggesting its complementary role in radiotherapy assessment. This case underscores the value of combining anatomic and functional imaging for evaluating radiotherapy outcomes and guiding clinical decision-making in feline oncology.

## 1 Introduction

Ceruminous gland adenocarcinoma is the most common malignant tumor of the feline external ear canal, which originates from modified apocrine glands. The tumor often exhibits locally invasive behavior and regional metastasis (1–3). Common clinical signs include otorrhea, head shaking, and pruritus; in advanced cases, local tumor extension may lead to facial nerve paralysis or vestibular dysfunction (3–5). Although surgical excision is typically the preferred treatment for ceruminous gland tumors, radiation therapy (RT) is considered a viable alternative when surgical access is limited or not feasible (3–5). Accurate assessment of tumor extent is essential for appropriate treatment planning. Computed tomography (CT) is well suited for evaluating osseous involvement, such as lysis of the tympanic bulla or skull base, whereas magnetic resonance imaging (MRI) offers superior soft tissue contrast, facilitating the detection of intracranial or perineural invasion (6–8).

Diffusion-weighted imaging (DWI) and apparent diffusion coefficient (ADC) mapping are functional MRI techniques increasingly applied in veterinary oncology to characterize tumor cellularity and monitor therapeutic response (7, 9, 10). In human studies, DWI has demonstrated high sensitivity and specificity for detecting post-treatment tumor recurrence and distinguishing it from post-radiation fibrosis (11). Additionally, studies in cats and dogs suggest that DWI may aid in detecting early post-radiation changes, particularly in brain and nasal tumors (7, 9, 10). Moreover, research in human head and neck cancers has demonstrated that early changes in ADC values during or post-RT can predict treatment response and long-term outcomes (12). Recent advancements in CT- and MRI-guided radiation planning have further improved precision while minimizing collateral damage to adjacent critical structures (7, 13, 14).

This case report presents the details of a feline ceruminous gland adenocarcinoma treated with palliative RT, with therapeutic response monitored using both CT and DWI MRI. To the authors’ knowledge, this represents a rare veterinary case illustrating the combined utilization of these imaging modalities to monitor extracranial tumor response.

## 2 Case description

**Figure 1:**
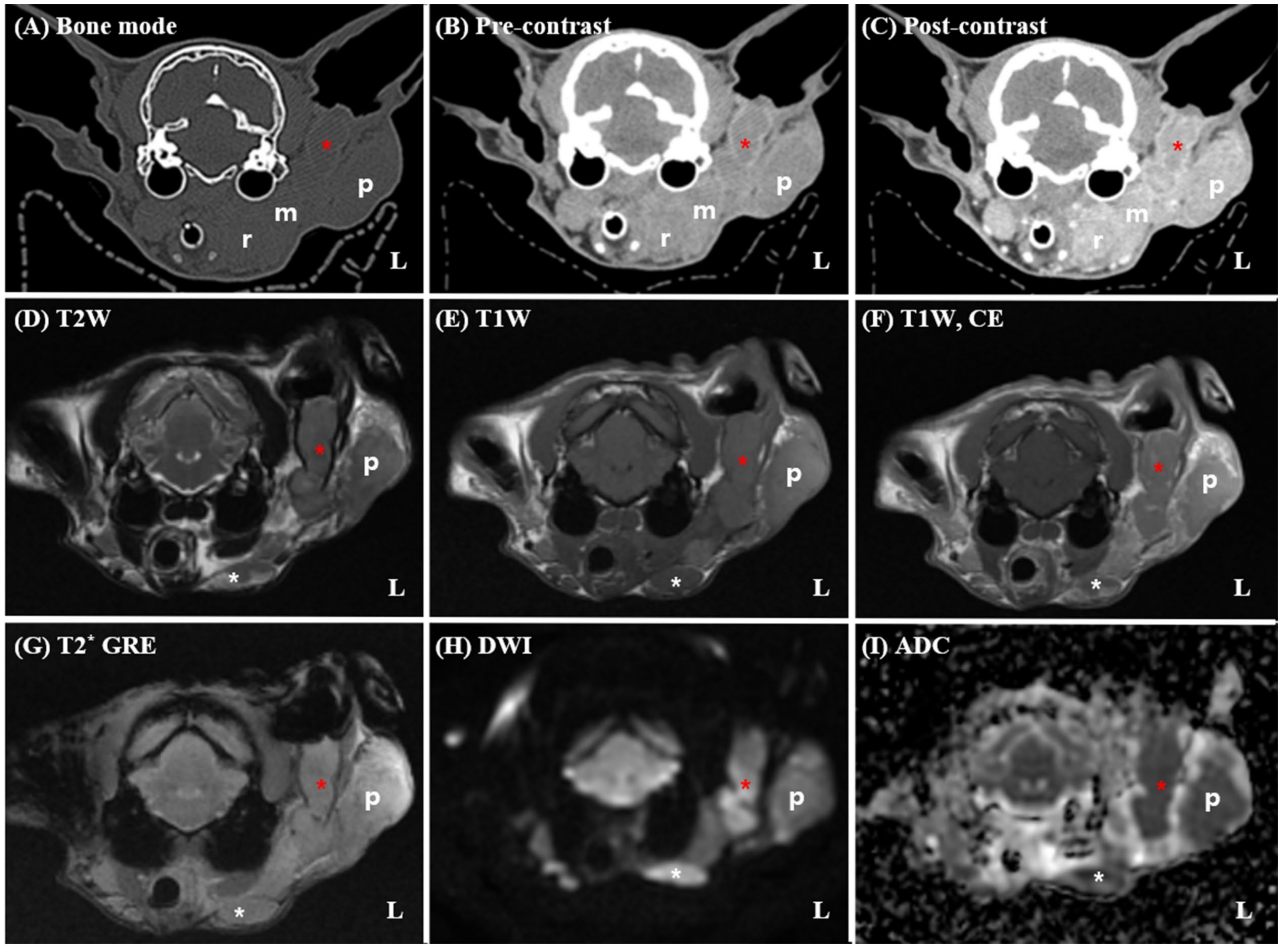
Pre-radiotherapy computed tomography (CT) **(A–C)** and magnetic resonance imaging (MRI) **(D–I)** findings in a 14-year-old spayed female Persian cat diagnosed with left ceruminous gland adenocarcinoma. The CT and MRI reveal a large soft tissue mass (red asterisks) occupying the left vertical external ear canal, with extension into the adjacent parotid (p) and mandibular regions. On post-contrast CT and T1-weighted MRI images **(C,F)**, the mass demonstrates strong, homogeneous enhancement. The margins between the mass and surrounding structures, including the masseter muscle and mandibular salivary gland (m), are indistinct, suggesting direct invasion. Marked enlargement of the ipsilateral mandibular (white asterisks) and medial retropharyngeal (*r*) lymph nodes are noted. The left lateral retropharyngeal lymph node is not clearly distinguishable from the primary mass, raising suspicion of direct tumor infiltration. Additionally, the left prescapular lymph node and parotid gland are severely enlarged (not shown). No evidence of osseous destruction or middle ear involvement is observed. On DWI **(G)**, the mass exhibits marked hyperintensity with corresponding hypointensity on the ADC map **(H)**, consistent with restricted diffusion and high cellularity. T1W, T1-weighted imaging; T2W, T2-weighted imaging; DWI, diffusion-weighted imaging; T1CE, post-contrast T1-weighted imaging; T2*, T2-weighted imaging; ADC, apparent diffusion coefficient.

A 14-year-old spayed female Persian cat weighing 3.09 kg was referred to Seoul National University for evaluation of a mass in the left external ear canal. The cat had previously been diagnosed with ceruminous gland adenocarcinoma based on an incisional biopsy performed at a local veterinary clinic and was undergoing medical treatment with toceranib phosphate (2.5 mg/kg PO). Despite treatment, the cat developed worsening pruritus in the left external ear canal and progressive enlargement of the mass. The general condition remained stable, with a normal appetite, urination, and defecation reported by the owner. The cat was bright, alert, and responsive during the examination and showed no signs of systemic illness. Upon physical examination, the cat exhibited a body condition score of 3/9, with mild dehydration noted based on skin turgor. Moderate brown, waxy discharge and a firm, irregular mass were detected within the left external ear canal, accompanied by mild swelling of the surrounding parotid region. The palpebral reflex was absent in the left eye, suggestive of facial nerve dysfunction, while other cranial nerve reflexes were intact. No enlargement of the submandibular or prescapular lymph nodes was palpated, and no vestibular or gait abnormalities were observed. Pain response during manipulation of the ear region was mild, and the cat tolerated the examination well. Thoracic radiographs revealed no evidence of pulmonary metastasis, and hematological and biochemical profiles were within normal limits. To assess tumor extent and plan radiation therapy, contrast-enhanced CT and MRI scans were performed under general anesthesia using an 80-row, 160-multislice CT scanner (Aquilion Lightning®; Canon Medical Systems Co., Otawara, Japan) and a 1.5-T MRI scanner (SIGNA Creator; GE Healthcare, Milwaukee, WI, USA) equipped with an 8-channel flex coil. Cross-sectional imaging revealed a large, extensive soft tissue mass occupying the left vertical ear canal, causing complete luminal obstruction and extending into the adjacent parotid and mandibular regions (Figure 1). The mass demonstrated strong, homogeneous enhancement on contrast-enhanced images. The left masticatory muscles and mandibular salivary gland exhibited similar enhancement patterns with indistinct margins, suggesting direct tumor invasion. DWI MRI showed marked restricted diffusion within the mass, characterized by hyperintensity on DWI and corresponding hypointensity on ADC maps, indicative of high cellularity. The mean ADC value of the mass was measured at 397 × 10^−6^ mm^2^/s. Multiple ipsilateral lymph nodes, including the mandibular, medial and lateral retropharyngeal, and prescapular lymph nodes, were markedly enlarged, homogeneously enhanced, and exhibited similar diffusion restriction, raising concerns for metastatic involvement. The right medial retropharyngeal lymph node was mildly enlarged. No evidence of pulmonary metastasis, osseous destruction, or abnormalities in the tympanic bullae, nasal cavities, or brain parenchyma was noted. Based on CT and MRI findings, the tumor demonstrated extensive local invasion into adjacent soft tissues, including the parotid region, mandibular salivary gland, and masticatory muscles, rendering surgical excision unfeasible.

**Figure 2:**
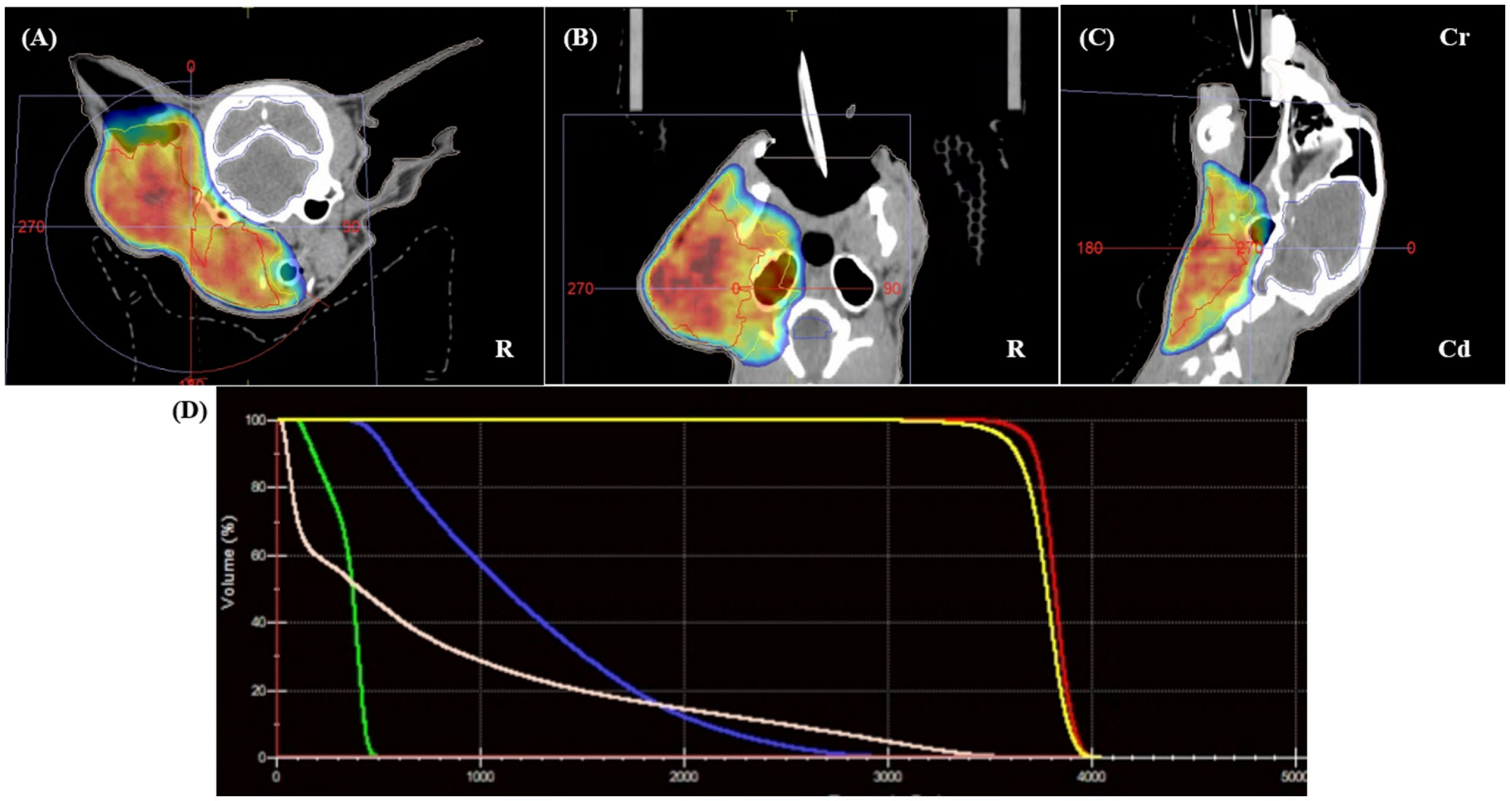
Radiotherapy planning using fused pre-treatment CT and MRI datasets. The gross tumor volume (GTV, red) includes the left ear canal mass, parotid and mandibular salivary glands, and enlarged ipsilateral lymph nodes. No clinical target volume (CTV) was applied. The planning target volume (PTV, blue) was generated by applying a 5-mm isotropic expansion to the GTV, encompassing suspected areas of microscopic tumor extension while avoiding unnecessary margins involving anatomical boundaries. Panels **(A–C)** show the delineated target volumes on transverse **(A)**, dorsal **(B)**, and sagittal **(C)** planes. Panel **(D)** displays the dose–volume histogram, demonstrating the distribution of the prescribed 36 Gy dose across the PTV, with dose constraints applied to organs at risk (OARs). Multimodal image fusion and Monte Carlo-based volumetric modulated arc therapy (VMAT) planning enabled precise dose coverage and sparing of adjacent critical structures.

Consequently, radiotherapy was chosen as the primary treatment modality. Based on image findings and client consultation, palliative radiation therapy was selected with the primary goal of alleviating clinical signs and reducing tumor size. For treatment simulation, the patient was positioned in sternal recumbency using a vacuum mattress (Vacuum Cushion, Chunsung Co., Seoul, Republic of Korea), thermoplastic mask (U-Frame, Chunsung Co., Seoul, Republic of Korea), and bite block. To improve anatomical delineation and target accuracy, fused CT and MRI datasets were used (Figure 2). The gross tumor volume (GTV) was defined as the contrast enhancing mass, including the left otic mass and ipsilateral parotid gland, mandibular gland, retropharyngeal lymph node, and submandibular lymph node. The clinical target volume (CTV) was generated by applying a 3 mm isotropic expansion to the GTV to encompass regions at risk of microscopic disease spread, including fluid in ear canal, peripheral edematous fat, and thickened fascia adjacent to the otic mass, while avoiding unnecessary margins in areas such as skin, body cavities, and anatomical boundaries. Planning target volume (PTV) was created by adding a 2 mm isotropic margin to the CTV. Organs at risk (OARs) were contoured, including the brain, spinal cord, cochlea, optic chiasm, optic nerves, eyes, lenses, lacrimal glands, salivary glands, and skin. A volumetric-modulated arc therapy (VMAT) protocol was planned; a total dose of 36 Gy in six weekly fractions of 6 Gy over 6 weeks was prescribed. RT planning was generated with the treatment planning system (Monaco version 6.1.2.0, Elekta, Stockholm, Sweden), which incorporates a Monte Carlo statistical algorithm. The primary plan objective was to achieve at least 98% of PTV was covered by 95% of the prescription dose. The maximum dose outside the PTV was limited to 107%. Two coplanar partial arcs were used, with beam collimation achieved using the MLC. Patient specific quality assurance (QA) was performed using dosimetric verification software (VeriSoft v8.1, PTW, Freiburg, Germany) and a detector array (OCTAVIUS Detector 1,500, PTW, Freiburg, Germany). QA parameters were set as a passing rate of 95% using 2 mm distance-to-agreement and 3% dose difference criteria, with an action limit of 90% and a threshold of 10%. After immobilizing the patient with the same positioning aids used for RT simulation, treatment was performed using a 6-MV photon linear accelerator (Elekta Synergy, Elekta AB, Stockholm, Sweden) equipped with a 160-micromultileaf beam collimator (Elekta Beam Modulator). Cone-beam CT imaging was performed prior to each treatment fraction for setup verification. RT was delivered as planned without incident.

During the early phase of radiotherapy, the patient developed mild erythema and purulent discharge around the nasal skin area, which improved significantly with regular cleaning and antiseptic care and had nearly resolved by the end of treatment (day 0). According to the Veterinary Radiation Therapy Oncology Group (VRTOG) criteria, this reaction was classified as a Grade 1 acute skin toxicity. No other significant adverse effects related to radiotherapy were observed. From day 0 to day 180, the cat maintained stable general health without recurrence of neurological signs or aural pruritus. Physical examination revealed a substantial reduction in the size of the mass throughout the follow-up period. Serial imaging assessments were conducted post-radiotherapy to evaluate treatment response and disease progression. Follow-up CT and MRI examinations were performed on day 0 (radiotherapy completion), and subsequently on days 30, 90, and 180, to monitor therapeutic response, evaluate residual disease, and assess changes in tumor dimensions, local invasion, and treatment-related complications.

**Figure 3:**
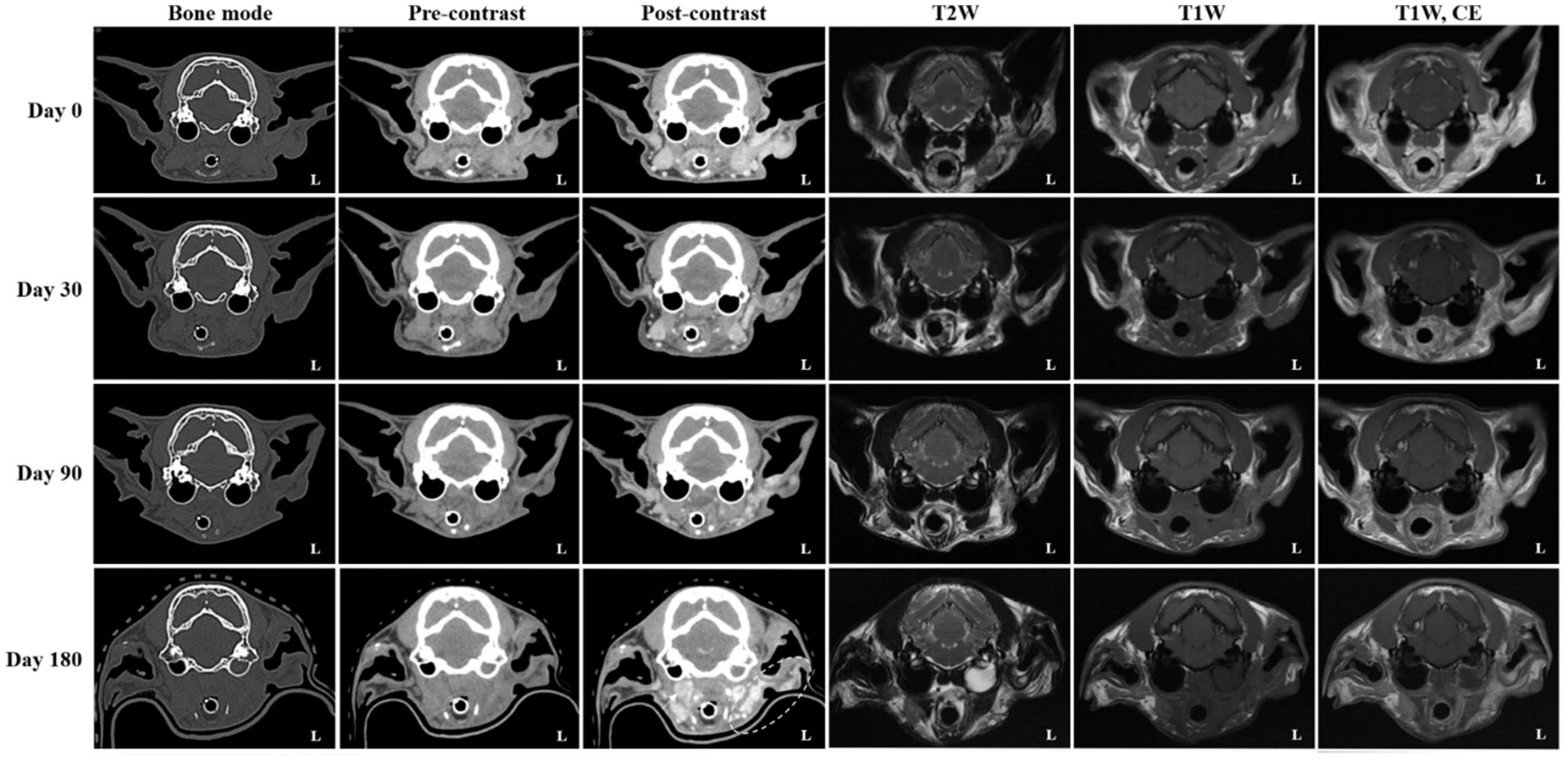
Serial computed tomography (CT) and magnetic resonance imaging (MRI) monitoring after palliative radiotherapy. Transverse post-contrast CT images obtained on days 0 (radiotherapy completion), 30, 90, and 180 illustrate temporal changes in the primary ear canal tumor and adjacent structures. Compared with pretreatment (baseline) images, the tumor showed more than a 50% reduction in maximal diameter at day 0, fulfilling the criteria for partial response (PR). By day 30, an additional ~30% reduction from baseline (approximately 80% total decrease) was observed, consistent with a continued PR. At day 90, only a minor further reduction (~10%) relative to the nadir was seen, maintaining a partial response (PR). By day 180, the lesion had increased by approximately 20% from the nadir and was accompanied by marked enlargement of the regional lymph nodes (arrow; left prescapular node not shown), meeting the criteria for progressive disease (PD). Additionally, fluid accumulation within the left tympanic cavity was evident on both CT and MRI, consistent with otitis media. The left parotid and mandibular regions (dotted circle) demonstrated increased contrast enhancement, raising suspicion for tumor progression or recurrence.

**Figure 4:**
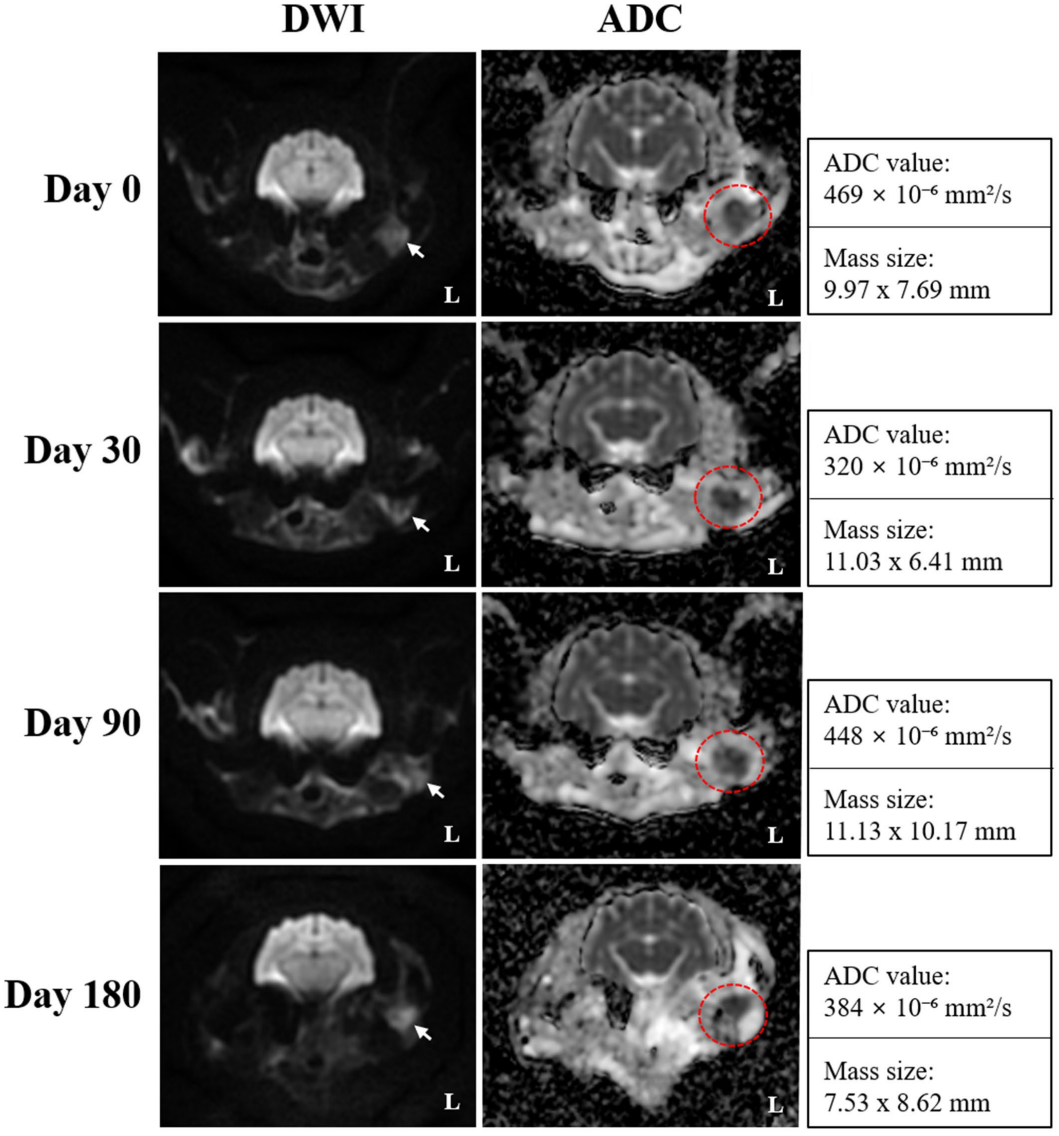
Serial diffusion-weighted imaging (DWI) and apparent diffusion coefficient (ADC) mapping obtained on days 0 (radiotherapy completion), 30, 90, and 180 for monitoring radiotherapy response in the present case. Transverse DWI and ADC images reveal a focal area of persistent diffusion restriction, which is characterized by hyperintensity on DWI (white arrows) and corresponding hypointensity on ADC maps (red dotted circles), and remained visible throughout the follow-up period, suggesting the presence of residual tumor tissue despite an overall favorable response. ADC mapping provided improved delineation of the lesion margins, and the persistently low ADC values indicated sustained high cellularity within the residual lesion.

On day 0 (radiotherapy completion), cross-sectional imaging demonstrated a significant reduction in the size of the mass in the left external ear canal (Figure 3). However, residual soft tissue remained within the external auditory canal, and although the previously invasive component extending into the left parotid and mandibular regions regressed markedly, it continued to exhibit strong contrast enhancement. The residual lesion displayed poorly defined margins and induced medial displacement and compression of the adjacent masseter muscle. Additionally, findings suggestive of concurrent otitis externa included thickening and calcification of the external ear canal wall, along with a small volume of luminal fluid. The left mandibular, medial retropharyngeal, and prescapular lymph nodes remained enlarged and rounded, although they were notably reduced in size compared to pre-treatment imaging. The right medial retropharyngeal lymph node remained mildly enlarged. MRI revealed hyperintense lesions on DWI and corresponding hypointense regions on ADC maps in the left mandibular and parotid regions, which visibly decreased in both extent and intensity (Figure 4). The mean ADC value and size of the mass were measured at 469 × 10^−6^ mm^2^/s and 9.97 × 7.69 mm, respectively. On day 30, further regression of the mass within the external auditory canal was noted on conventional CT and MRI.

Nevertheless, contrast enhancement and indistinct margins persisted in the left parotid and mandibular regions. The mandibular and retropharyngeal lymph nodes continued to show a reduction in size, with only mild enlargement remaining. However, the left prescapular lymph node was notably enlarged compared to the previous scan. On DWI and ADC imaging, the signal intensity and size of hyperintense lesions on DWI remained similar to those on day 0. The mean ADC value and size of the mass was calculated at 320 × 10^−6^ mm^2^/s and 11.03 × 6.41 mm, respectively. On day 90, no significant changes in the size, extent, or imaging characteristics of the residual lesion were observed compared to day 30. Severe enlargement of the left prescapular lymph node, along with the mandibular, medial, and lateral retropharyngeal lymph nodes, remained similar. On DWI and ADC imaging, hyperintense lesions on DWI also remained stable. The mean ADC value and size of the mass were recorded at 448 × 10^−6^ mm^2^/s and 11.13 × 10.17 mm, respectively. Persistent findings in the left parotid and mandibular regions suggested the presence of residual neoplastic tissue.

On day 180, although the size and extent of the residual mass on conventional CT and MRI showed no remarkable changes, there was marked disease progression, characterized by marked enlargement of multiple lymph nodes, including the left medial retropharyngeal, left prescapular, and right medial retropharyngeal nodes. Furthermore, contrast enhancement in the left parotid and mandibular regions increased, indicating potential tumor recurrence or progression. Concurrent left-sided otitis media was newly identified. The mean ADC value and size of the mass was measured at 384 × 10^−6^ mm^2^/s and 7.53 × 8.62 mm, respectively. Based on serial CT and MRI evaluations, the tumor response in this case was classified according to the Veterinary Cooperative Oncology Group (VCOG) response evaluation criteria for solid tumors in dogs (v1.0) (15), using the maximal diameter of the primary lesion as the measurement parameter. Compared with the pretreatment baseline, the tumor size at day 0 showed more than a 50% reduction, fulfilling the criteria for partial response (PR). By day 30, an additional 30% decrease relative to day 0 was observed, resulting in an overall reduction of approximately 80% compared with baseline, consistent with a continued PR. At day 90, the lesion showed minimal further reduction (~10%) compared with the nadir, maintaining a partial response (PR). By day 180, the lesion had increased by approximately 20% from the nadir, accompanied by marked enlargement of regional lymph nodes, meeting the criteria for progressive disease (PD).

## 3 Discussion

In this case, surgical excision was deemed unfeasible due to the tumor’s extensive local invasion into adjacent structures, including the mandibular salivary gland and masticatory muscles, as identified on CT and MRI (3–5). The tumor exhibited strong contrast enhancement with indistinct margins and restricted diffusion on DWI, suggestive of high cellularity and malignancy (13, 16). Enlarged ipsilateral lymph nodes displayed similar imaging features, raising concerns for metastatic involvement (16). Given the anatomical complexity and high surgical risk, radiation therapy was chosen as the primary treatment modality (3–5). VMAT was employed to enable conformal dose delivery while minimizing radiation exposure to surrounding critical tissues (12).

Surgical excision, such as total ear canal ablation with lateral bulla osteotomy, is the standard treatment for ceruminous gland adenocarcinoma when feasible, offering the best chance for local control (3–5). However, in cases with extensive local invasion or advanced-stage disease, complete resection may not be possible. Palliative-intent radiotherapy has been utilized as an alternative, particularly when surgery is impractical. Although median survival times (MSTs) are typically shorter than those achieved with surgery, radiotherapy can yield partial tumor regression and symptomatic relief, with reported MSTs ranging from 3 to 10 months (11, 13). Surgical complications may include facial nerve paralysis or chronic otitis, whereas radiotherapy, particularly advanced techniques like VMAT, tends to be associated with lower morbidity and manageable side effects (12–14).

Although this case eventually indicated evidence of tumor progression and nodal metastasis on day 180, the treatment can still be considered relatively successful within the context of palliative radiotherapy. The primary treatment objectives were tumor size reduction and symptomatic relief, which were largely achieved. Following RT, tumor volume and lymph node enlargement regressed significantly, and the cat exhibited stable general health, remaining free of neurological signs or pruritus during the 6-month follow-up period. Mild and transient radiation-associated adverse effects resolved with supportive care. These findings indicate that palliative-intent radiotherapy effectively controlled clinical signs and enhanced quality of life, even during this advanced, non-resectable case. This case supports the potential utility of palliative VMAT as a viable and safe treatment option for feline ceruminous gland adenocarcinoma when surgical excision is not feasible.

The combined use of contrast-enhanced CT and functional MRI, encompassing DWI and ADC mapping, proved crucial in monitoring post-radiotherapy response. Although CT provided insights into tumor size and anatomical alterations, DWI and ADC sequences facilitated a more sensitive evaluation of residual viable tissue, especially when conventional sequences yielded ambiguous results (13, 14, 16). DWI effectively highlights areas of high cellularity through hyperintensity, whereas ADC maps represent these regions as hypointense due to restricted water diffusion. In this case, residual areas of restricted diffusion persisted from day 0 to day 180; however, lesion size and ADC values exhibited minimal overall change during this timeframe (469 → 384 × 10^−6^ mm^2^/s), indicating enduring tumor tissue with stable characteristics. These observations suggest that ADC mapping was useful in delineating residual lesion margins and assessing ongoing tumor burden, even in the absence of dramatic morphologic changes. This finding aligns with reports in human oncology, wherein functional MRI alterations often precede anatomical changes and assist in predicting outcomes (14, 16). This case highlights the potential utility of DWI/ADC sequences for treatment response monitoring in extracranial feline tumors, which is an area rarely documented in veterinary literature (7, 9, 10).

On day 180, newly progressive changes–including enlargement of the contralateral medial retropharyngeal lymph node, increased enhancement in the mandibular and parotid regions, and concurrent left otitis media–suggested tumor recurrence and metastasis. Although these findings imply a guarded prognosis moving forward, the outcome can still be interpreted as a relatively favorable palliative response, particularly when compared to MSTs reported in prior studies of feline ceruminous gland carcinomas (2–6). Despite signs of disease progression, the patient remained clinically stable and free of neurologic symptoms throughout the follow-up period, with initial palliative radiotherapy goals achieved. Thus, this case underscores the importance of balancing imaging findings with clinical outcomes in evaluating treatment success. Continued monitoring beyond 6 months is essential to reassess prognosis, and serial ADC mapping may serve as an early indicator of recurrence prior to overt morphological changes occurring.

This study has several limitations. First, as a single case report, the findings cannot be generalized without caution. Although the therapeutic course and imaging follow-up provide valuable clinical insight, broader validation from larger populations is necessary to support definitive conclusions. Second, the follow-up period was limited to 6 months, which may not have been adequate to evaluate long-term tumor control, recurrence, or late-onset radiation-related complications comprehensively. Longer-term monitoring would enhance understanding of the durability of the treatment response. Third, histopathological or cytological confirmation of suspected metastatic lymph nodes or residual/infiltrative lesions was not obtained because the owner declined further invasive procedures due to the palliative intent of treatment. Consequently, interpretations regarding regional metastasis and post-treatment residual disease were based solely on imaging features, which are suggestive yet remain presumptive. This limitation may have influenced staging accuracy and treatment evaluation, and should be taken into account when interpreting the clinical outcome of this case. Fourth, interpretation of DWI and ADC mapping poses inherent technical limitations. ADC values may be affected by the inclusion of necrotic or cystic areas, potentially underestimating true cellularity. In this case, the region-of-interest (ROI) placement was manually performed; efforts were made to avoid non-solid components, however some heterogeneity cannot be excluded (13, 14, 16). Finally, the spatial resolution of DWI/ADC sequences is inherently lower than that of conventional sequences, which may have diminished the accuracy of small lesion delineation or subtle tissue interface evaluation (13, 144, 16). Despite providing useful adjunctive information, consistently low ADC values over time did not correspond with tumor progression observed on day 180, which may limit their predictive utility in specific cases. Nonetheless, DWI and ADC contributed valuable additional information for evaluating treatment response and residual disease, reinforcing their complementary role in post-RT monitoring.

This report presented a rare case that demonstrated the use of palliative radiotherapy combined with CT and functional MRI, including DWI and ADC mapping, to monitor therapeutic response in a feline with ceruminous gland adenocarcinoma. The findings highlight the clinical value of advanced imaging in assessing post-treatment residual disease, particularly when surgery is not feasible. Although the disease ultimately progressed by day 180, the case illustrated how palliative-intent radiotherapy can provide meaningful clinical benefits, including symptomatic relief, tumor burden reduction, and maintenance of patient condition. This approach may provide a new direction for radiologic assessment of treatment response and prognosis prediction in veterinary oncology. Additionally, the fusion of CT and MRI facilitated more accurate target delineation and response evaluation, emphasizing the utility of multimodal imaging strategies in radiation therapy planning and follow-up.

## Data Availability

The datasets presented in this study can be found in online repositories. The names of the repository/repositories and accession number(s) can be found in the article/supplementary material.
